# A distributional regression approach to income-related inequality of health in Australia

**DOI:** 10.1186/s12939-020-01189-1

**Published:** 2020-06-22

**Authors:** Roselinde Kessels, Anne Hoornweg, Thi Kim Thanh Bui, Guido Erreygers

**Affiliations:** 1 Department of Data Analytics and Digitalization, Maastricht University, PO Box 616, Maastricht, 6200 MD The Netherlands; 2 Department of Economics, University of Antwerp, City Campus, Prinsstraat 13, Antwerp, 2000 Belgium; 3 School of Economics, University of Amsterdam, PO Box 15867, Amsterdam, 1001 NJ The Netherlands; 4 School of Economics, Can Tho University, Campus II, 3/2 Street, Can Tho City, Vietnam; 5 Centre for Health Policy, University of Melbourne, Bouverie Street 207, Carlton, Victoria, 3010 Australia

**Keywords:** Socioeconomic health inequality, Distributional regression, GAMLSS, Quantile regression

## Abstract

**Background:**

Several studies have confirmed the existence of a significant positive relationship between income and health. Conventional regression techniques such as Ordinary Least Squares only help identify the effect of the covariates on the mean of the health variable. In this way, important information of the income-health relationship could be overlooked. As an alternative, we apply and compare unconventional regression techniques.

**Methods:**

We adopt a distributional approach because we want to allow the effect of income on health to vary according to people’s health status. We start by analysing the income-health relationship using a distributional regression model that falls into the GAMLSS (Generalized Additive Models for Location, Scale and Shape) framework. We assume a gamma distribution to model the health variable and specify the parameters of this distribution as linear functions of a set of explanatory variables. For comparison, we also adopt a quantile regression analysis. Based on predicted health quantiles, we use both a parametric and a non-parametric approach to estimate the lower tail of the health distribution.

**Results:**

Our data come from Wave 13 of the Household, Income and Labour Dynamics in Australia (HILDA) survey, collected in 2013-2014. According to GAMLSS, we find that the risk of ending up in poor, fair or average health is lower for those who have relatively high incomes ($80,000) than for those who have relatively low incomes ($20,000), for both smokers and non-smokers. In relative terms, the risk-lowering effect of income appears to be the largest for those who are in poor health, again for both smokers and non-smokers. The results obtained on the basis of quantile regression are to a large extent comparable to those obtained by means of GAMLSS regression.

**Conclusions:**

Both distributional regression techniques point in the direction of a non-uniform effect of income on health, and are therefore promising complements to conventional regression techniques as far as the analysis of the income-health relationship is concerned.

## Background

Whether we look at regional, national or even global data, we always find that income and health are unequally distributed: some of us are rich, while others are poor; some of us live long and healthy lives, while others suffer and die young. We also know that income and health tend to be positively correlated, in such a way that higher income levels are often associated with better health outcomes, a phenomenon often referred to as the ‘social gradient’. Yet, the exact nature of the income-health relationship is complex. A lack of income reduces the options to lead a healthy lifestyle, and therefore constitutes an important determinant of the often observed social gradient in healthy behaviours [1]. It also acts as a barrier for access to health care, which may be conducive to bad health. Even in countries that have universal health care coverage, such as Australia, affordability remains a barrier for access to health care [2]. In reverse, bad health may be a factor contributing to job loss and therefore lead to low income [3].

Econometric research on this issue is often based on conventional regression techniques, which focus on the explanation of the mean, i.e. the expected value, of the dependent variable. In this paper, we explore and compare two alternative regression techniques, which allow for the possibility that the income-health relationship differs according to the location in the distribution, e.g. different for those who are in good health and for those who are in bad health. More specifically, we will use a recently developed ‘distributional’ regression technique in the form of generalized additive models [4] as well as the increasingly popular quantile regression method [5]. Our aim with this paper is to get a better understanding of the full spectrum of the income-health relationship, rather than trying to measure the social gradient by means of an index number (e.g., [6]) or to explain it by means of a regression-based decomposition (e.g., [7]).

Based on a variety of income measures and health variables, several studies have confirmed the existence of a significant positive relationship between income and health (e.g., [8–11]). As pointed out by Silbersdorff et al. [12], the majority of studies use linear and generalized linear models, which assess the effect of variations in independent variables on the expected level of the dependent variable. With regard to the income-health relationship, this means that variations in income are solely related to the conditional mean health outcome. In this way, important information of the income-health relationship could be overlooked. Although the general trend of the relationship might be captured, aspects such as differences in variance or skewness in the distribution are neglected.

To account for the fact that the expectation of a variable is not necessarily representative of its entire distribution, the focus in income-health relationship studies has shifted to unconventional regression approaches. Unconventional regression methods provide a more complete picture of distributional characteristics, since these methods look at effects beyond the mean. However, the amount of studies exploring these unconventional regression methods is still limited, and they mostly focus on one specific regression technique only. In this paper, we employ two unconventional regression techniques to examine the income-health relationship, using Australian data collected in 2013-2014. We study the conditional health distribution by means of the distributional regression method designated by the acronym GAMLSS, which stands for generalized additive models for location, scale and shape [4], and by means of quantile regression [5]. We are especially interested in the effect of income on the probabilities of ending up in bad health.

### The income-health relationship

The direction of causality constitutes an important issue in the literature on income and health, with wide-ranging implications for public policies. One strand of this literature studies the relationship at the country level, inspired by the work of Preston [13] and Deaton [14]. The famous Preston curve, representing a positive, concave relationship between GDP (Gross Domestic Product) per capita and life expectancy, strongly suggests that increases in average income are among the driving forces of increases in population health. Others have looked at the relationship between per capita income and per capita health expenditures. Erdil and Yetkiner [15], for instance, adopted a panel data regression approach to study this relation. Using Granger causality tests, they found that in most cases the causal relationship is bidirectional. When they found one-way causality, it tended to be from GDP to health expenditures in low- and middle-income countries, but the other way around in high-income countries. Another area of cross-country research is about the relationship between income inequality and population health. Babones [16], for instance, claimed that the evidence for the existence of a strong, statistically significant causal relation between income inequality and health is rather weak. By contrast, based on an extensive literature study and nine criteria for causality, Pickett and Wilkinson [11] argued that as far as developed countries are concerned the available evidence strongly indicates the existence of a causal relation between income inequality and health: high levels of income inequality lead to low levels of health (e.g., life expectancy).

However, most of the literature is about the relation between income and health at the individual level, and this is also what we will be focusing on in this paper. As far as Australia is concerned, several studies have used survey data to estimate the social gradient of health by means of the concentration index and to compare Australia to other countries [17–19]. Various decomposition techniques have been applied in order to come to a better understanding of the underlying mechanisms [7, 19, 20]. For other countries, more complex econometric methods have been tried. A good example is the study by Frijters, Haisken-DeNew and Shields [21], who looked at the effect of household income on individual health satisfaction in East Germany, using a fixed-effects ordered logit model. Even though they found some evidence for a positive effect, they emphasized that it was very small. In another study, Kuehnle [22] focused on the relation between household income and child health, choosing an instrumental variable (IV) approach to control for the potential endogeneity of income. He too arrived at the conclusion that income has a positive effect on health, albeit a very small one. These studies illustrate that the nature of the relationship is complex: income influences health, health influences income, and other factors influence both income and health. Advanced econometric techniques are required to disentangle the multiple aspects of the income-health relationship, and even then clear-cut statements about causality may be difficult to obtain.

Most empirical studies on the effect of income on health have used ordinary least square and IV approaches, generalized linear models, and comparisons of correlation coefficients [8–10]. However, Silbersdorff et al. [12] argue that conventional regression techniques do not provide reliable estimates of the income-health relationship. Conventional regression techniques assess the effect of variations in covariates on the expected level of the dependent variable. Following a different approach, Silbersdorff et al. [12] show that in Germany the effect of income on health is not uniform throughout the whole distribution. In particular, relatively high probabilities of outcomes in the left tail of the health distribution are observed for the part of the sample with low income levels. This cannot be revealed by conventional regression methods.

In this paper, we study the income-health relationship by means of two alternative regression techniques, GAMLSS and (conditional) quantile regression, which both estimate conditional distribution models. One of our aims is to investigate whether the results obtained by these models are similar. A comparison with other recently developed regression techniques, such as unconditional quantile regression, also known as recentered influence function (RIF) regression [23], is left to future research.

## Methods

### GAMLSS

One way to examine a more complete health distribution is via GAMLSS, as introduced by Rigby and Stasinopoulos [4]. Relatively to mean regression techniques, GAMLSS aim to describe the full conditional distribution of the dependent variable by estimating not only the mean but also other distributional characteristics such as variance and skewness. When a regression of health is conducted via GAMLSS, all parameters of the health distribution are linked to a set of explanatory variables. In their paper, Hohberg, Pütz and Kneib [24] provide a clear guidance into GAMLSS. If we consider a population of *N* individuals ($i = 1,\dots, N$) and a health variable *h*, GAMLSS assume that the observed *h*_*i*_ are conditionally independent and described by a parametric distribution:

1 $$ f(h_{i}|y_{i},X_{i}) = f(h_{i}|\theta_{1}(y_{i},X_{i}),\dots,\theta_{L}(y_{i},X_{i}))  $$

where $\theta _{1},\dots,\theta _{L}$ are *L* different parameters of the distribution that are conditional on income *y*_*i*_ and other socioeconomic variables contained in the vector *X*_*i*_. Each parameter *θ*_*l*_, $l = 1,\dots, L$, is connected to a regression predictor $\eta ^{\theta _{l}}\phantom {\dot {i}\!}$, also conditional on *y*_*i*_ and *X*_*i*_, via a link function *g*_*l*_ such that $\theta _{l} = g_{l}^{-1}(\eta ^{\theta _{l}})$. In our setting, the predictor function takes on the following additive form:

2 $$ \eta^{\theta_{l}}(i) = \beta_{0}^{\theta_{l}} + \beta_{1}^{\theta_{l}} y_{i} + \beta_{2}^{\theta_{l}} X_{i}   $$

where $\beta _{0}^{\theta _{l}}$, $\beta _{1}^{\theta _{l}}$ and $\beta _{2}^{\theta _{l}}$ denote the regression coefficients for predicting *θ*_*l*_. By choosing an appropriate link function, it can be ensured that restrictions of the parameter space are fulfilled (e.g., a log link to ensure positive standard deviations). After having selected the distribution, predictor setup and link functions, the unknown regression coefficients can be estimated by maximizing a penalized likelihood function in a classical frequentist approach [4] or by Bayesian methods [25].

The use of GAMLSS requires the specification of a parametric distribution that approximates the observed health outcomes. In our application described in the Results section, we use a continuous health score, the distribution of which is negatively skewed. As stated in [12] and [26], continuous health distributions generally exhibit a negative skewness, whereas most parametric specifications are suited for the more common symmetric or positively skewed distributions. To be able to work with a health distribution that is positively skewed, we linearly transform the health score *h* as follows:

3 $$ h^{*} = \frac{h_{0} - h}{h_{scale}}   $$

where *h*_0_ is a constant ensuring that the transformed health score *h*^∗^ has positive support and *h*_*scale*_ is a rescaling factor. The health score in our application is a bounded variable on the unit interval [ 0,1]. To obtain positive skewness in the distribution, we use *h*_0_=1.0001 and *h*_*scale*_=1, so that transformation (3) becomes:

4 $$ h^{*} = 1.0001 - h   $$

where *h*^∗^ is restricted to the interval [ 0.0001,1.0001]. As recommended by Silbersdorff et al. [12] and Silbersdorff and Schneider [26], we use the two-parameter gamma distribution to approximate the transformed health score. This distribution provides a sufficiently good fit compared to other distributions, such as the two-parameter Weibull and lognormal distributions, as we show in Appendix 1. We obtain the conditional distribution of the untransformed health score by calculating the inverse transformation. Note that the two-parameter gamma distribution for modelling the transformed health score is bound by zero, *h*^∗^>0, and positively skewed. Given the location parameter *μ*>0 and the scale parameter *s*>0, the distribution can be written as

5 $$ f(h^{*}|\mu,s) = \frac{h^{*^{\frac{1}{s^{2}}-1}}\text{exp}({-h^{*}/(s^{2}\mu)})}{(s^{2}\mu)^{(1/s^{2})}\Gamma \left(1/s^{2}\right)}  $$

where *Γ* denotes the gamma function. Also, *E*(*h*^∗^)=*μ* and *Var*(*h*^∗^)=*σ*^2^=*s*^2^*μ*^2^. This expression of the gamma distribution corresponds to the GA formulation provided by Rigby et al. [27] (see p. 271).

Because we focus on the gamma distribution with parameters *μ* and *s*, the corresponding link functions are logarithmic to ensure the positivity of the two parameters. Also, in our application, the functional form of the predictors for the two parameters is the same as in Eq. (2). Hence, the predictor setups and link functions are as follows:

6 $$ log(\mu) = \beta_{0}^{\mu} + \beta_{1}^{\mu} y + \beta_{2}^{\mu} X   $$

7 $$ log(s) = \beta_{0}^{s} + \beta_{1}^{s} y + \beta_{2}^{s} X   $$

Because we adopt a standard modelling approach that is similar to a previous application in [26], we follow the frequentist estimation framework provided by the GAMLSS package [28] in the statistical software R 3.6.2 [29]. We refer the reader interested in a Bayesian implementation of GAMLSS that can deal with more advanced modelling situations to applications in [30] and [12].

### Quantile regression

Another regression approach that goes beyond the mean is quantile regression [31]. In quantile regression, the estimation of a conditional mean function is replaced by estimations of different conditional quantile functions. Koenker and Hallock [5] define conditional quantile functions as models that express quantiles of the conditional distribution of the dependent variable as functions of independent variables. For our health variable *h*, characterized by its cumulative distribution function (CDF), *F*_*h*_(*h*_*j*_)=*P*(*h*≤*h*_*j*_), the *τ*th quantile, 0<*τ*<1, is defined as

8 $$ h^{\tau} = \inf\{h_{j}: F_{h}(h_{j}) \geq \tau\}  $$

Alternatively, the *τ*th quantile is defined as the solution satisfying the inequalities:

9 $$ h^{\tau} \leq h_{j} \quad \text{if and only if} \quad F_{h}(h_{j}) \geq \tau   $$

To understand the income-health relationship, a quantile regression approach is used to describe the entire conditional distribution of *h*_*i*_ by quantile functions of income *y*_*i*_ and a set of control variables *X*_*i*_ in a form similar as in Eq. (2):

10 $$ h_{i}^{\tau}(y_{i}, X_{i}) = \beta_{0}^{\tau} + \beta_{1}^{\tau} y_{i} + \beta_{2}^{\tau} X_{i} + \epsilon_{i}^{\tau}   $$

where $\beta _{0}^{\tau }$, $\beta _{1}^{\tau }$ and $\beta _{2}^{\tau }$ denote the regression coefficients for the *τ*th quantile and $\epsilon _{i}^{\tau }$ is the error term.

The conditional quantile functions (10) are estimated in the same manner as the conditional mean function in least squares regression. However, instead of minimizing the sum of squared residuals over a sample of *N* observations, in quantile regression a sum of asymmetrically weighted absolute residuals is minimized:

11 $$ \begin{aligned} &{\min_{\beta_{0}^{\tau}, \beta_{1}^{\tau}, \beta_{2}^{\tau}} \sum\limits_{i=1}^{N} \rho_{\tau}\left(h_{i} - (\beta_{0}^{\tau} + \beta_{1}^{\tau} y_{i} + \beta_{2}^{\tau} X_{i})\right)}\ \\ &\text{with}\ \rho_{\tau}(u) = \left\{ \begin{array}{ll} u\tau & \text{for}\ u\ \geq\ 0\\ u(\tau - 1) & \text{for}\ u\ <\ 0\end{array} \right.  \end{aligned}  $$

The function *ρ*_*τ*_(*u*) is called the check function which is a loss function that retrieves the *τ*th health quantile. Since we assume that *h* is linear in the regression coefficients, the minimization problem can be solved efficiently via linear programming methods. We use the implementation in the R package quantreg [32] to obtain the estimated coefficients. For given values of *y* and *X*, the predicted health quantile $\hat {h}^{\tau }$ can then be identified as

12 $$ \hat{h}^{\tau}(y,X) = \hat{\beta}_{0}^{\tau} + \hat{\beta}_{1}^{\tau}y + \hat{\beta}_{2}^{\tau}X  $$

In our application, we focus specifically on the value of $\hat {\beta }_{1}^{\tau }$, which is the estimated marginal effect of income on health, given that the observation is, and remains in, quantile *τ*.

### Comparison of quantile regression to GAMLSS

To compare quantile regression to GAMLSS, we consider an individual with given values for *y* and *X*. The GAMLSS approach estimates the parameters of the corresponding gamma distribution. The quantile regression approach provides the predicted health quantiles $\hat {h}^{\tau _{k}}(y,X)$ for different values of *τ*_*k*_, $k = 1,\dots,K$. We can use these predicted quantiles to estimate the parameters of a gamma distribution by sorting them in an empirical CDF. The CDF of a gamma distribution is continuous and strictly monotonically increasing, such that the inequalities in expression (9) can be replaced by equalities. Assuming a gamma distribution, the predictions $\hat {h}^{\tau _{k}}(y,X)$ thus result in *K* estimated values of the theoretical CDF:

13 $$ F_{h}(\hat{h}^{\tau_{k}}) = P(h \leq \hat{h}^{\tau_{k}}) = \tau_{k} \quad \text{for}\ k = 1,\dots,K  $$

Inversely, $\hat {h}^{\tau _{k}} = F^{-1}_{h}(\tau _{k})$.

Because in our application the gamma distribution is used to model the transformed health score *h*^∗^=1.0001−*h*, we estimate the theoretical CDF from the transformed values of the predicted health quantiles $\widehat {h}^{*}_{k} = 1.0001-\hat {h}^{\tau _{k}}$, as given by

14 $$ P(h^{*} \leq \widehat{h}^{*}_{k}) = 1 - \tau_{k} \quad \text{for}\ k = 1,\dots,K  $$

We obtain the two parameters of the corresponding gamma distribution, *μ* and *s*, by minimizing the sum of squared residuals between the empirical and theoretical CDFs:

15 $$ \min_{\mu,s} \sum\limits_{k=1}^{K} \left(1 - \tau_{k} - F_{h^{*}}\left(\widehat{h}^{*}_{k},\mu,s\right)\right)^{2}  $$

where $F_{h^{*}}(\widehat {h}^{*}_{k},\mu,s)$ is the theoretical CDF of the gamma distribution for $\widehat {h}^{*}_{k}$. We can now compare the estimated gamma distribution from quantile regression to the one directly obtained from GAMLSS.

## Results

We compare the two distributional regression methods, GAMLSS and quantile regression, for analysing the income-health relationship in an application using Australian data. We first describe these data after which we report on the results.

### Data description

We examine the income-health relationship in Australia using data from the Household, Income and Labour Dynamics in Australia (HILDA) survey. This survey is a nationally representative household-based panel study, containing observations on individuals aged 15 years or older. Since the start of the survey in 2001, it is repeated annually with the aim to follow the same group of residents over the course of their lives. The survey includes questions about income and employment, household and family relationships, and personal well-being. Similarly as in a previous study [7], data from Wave 13, collected in the years 2013-2014, are used. In total, the sample consists of 14,728 individuals. In what follows, we provide a description of the variables retained for analysis. We begin with our response variable, the health score, followed by our main independent variable, income, after which we highlight the control variables.

**Fig. 1:**
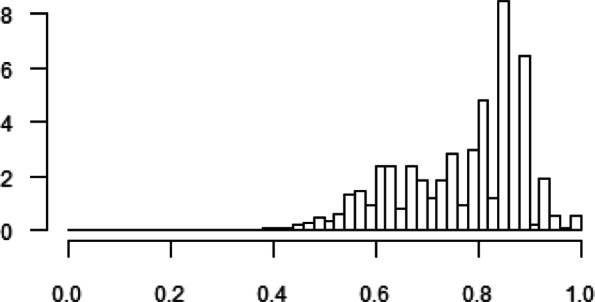
Histogram of the SF-6D health score

To be able to conduct standard quantile regression, a continuous response variable is required. We use the SF-6D health score, which is a health state classification composed of six health dimensions. It is a preference-based single index measure of health, bounded on the unit interval [0, 1], that can be used for economic evaluations [33]. Figure 1 shows the histogram of the SF-6D (Short-Form Six-Dimension) health score, which is negatively skewed. Silbersdorff et al. [12] and Silbersdorff and Schneider [26] argued that continuous health measures typically have negatively skewed distributions.

The main explanatory variable of interest is the logarithm of income, and in particular equivalized income, which is commonly used in similar research [12, 34]. Equivalized income is calculated using the OECD-modified equivalence scale. This scale assigns an equivalence factor to each household type in proportion to the household’s needs. The equivalence factor depends on the size of the household and the ages of its members. A value of 1 is assigned to the first adult of the household, a value of 0.5 to each additional adult and a value of 0.3 to every child. The equivalence factor is the sum of these values. Dividing the household’s disposable income by the equivalence factor equals the equivalized income for each household member.

In addition to income, we included a set of control variables in the regressions that turned out to affect the health outcomes. Typical variables that emerge from the existing literature on income and health are gender, age, ethnicity, occupational class, marital status and the number of children [8, 9, 21, 35]. We incorporated all these variables except gender and marital status because they were not significant in the estimation of the gamma distribution via GAMLSS. That is to say, gender and marital status had almost no discernible effect on the health score distribution, neither on their own nor in interaction with income or any other covariate. Instead, we added several other variables to the analysis that significantly improved the estimation of the gamma distribution. These variables describe lifestyle and individual health characteristics: sleep quality, physical activity, smoking, time stress, life satisfaction and satisfaction with weight.

A complete list of the variables used and their descriptive statistics are presented in Table 1. We treat the variables income, age, number of children aged 0-4 years and aged 5-14 years, and life satisfaction as numerical. We also specify an individual’s age nonlinearly in the regression models using a squared term that we mean-center to remove multicollinearity with the linear term. All other variables are categorical and enter into the regressions as dummy variables. In this respect, we define the reference person as a non-indigenous person who provides a professional service, has a fairly good sleep quality, does frequent physical activity, does not smoke, is sometimes stressed for time and is neither satisfied/dissatisfied with one’s weight.

**Table 1:** Descriptive statistics of the sample (*N*= 14,728)

**Numerical variables**	**Mean**	**S.D.**	**Min**	**Max**
SF-6D health score	0.76	0.12	0.3	1
(Equivalized) income ($1000)	51.13	34.92	0.001	781.22
Age	44.87	18.57	15	96
Age squared: (Age−44.87)^2^	344.92	368.09	0.02	2614.69
Number of children				
Aged 0-4	0.18	0.51	0	4
Aged 5-14	0.30	0.71	0	7
Life satisfaction (0, 1,..., 10)	7.93	1.41	0	10
**Categorical variables (in %, with reference indicated in bold)**				
Ancestry		Smoking		
Indigenous	2.50	Yes		17.57
**Non-indigenous**	97.50	**No**		82.43
Occupational status		Time stress		
Managers & professionals	24.57	Almost always		8.73
**Service & sales workers**	29.87	Often		26.76
Manual workers	9.02	**Sometimes**		40.89
Unemployed	4.04	Rarely		20.77
Not in labour force	32.50	Never		2.85
Sleep quality		Satisfaction with weight		
Very good	19.39	Very satisfied		8.98
**Fairly good**	54.97	Satisfied		26.69
Fairly bad	20.48	**Neither satisfied/**		24.19
		**dissatisfied**		
Very bad	3.97	Dissatisfied		31.47
Not reported	1.19	Very dissatisfied		8.67
Physical activity				
No	11.11			
Some	37.27			
**Frequent**	51.62			

### GAMLSS results

The estimated GAMLSS regression model generates coefficients for the predictor functions (6) and (7) with which we can obtain gamma distributions for different types of individuals. More specifically, we use these gamma distributions to estimate the effect of income on the probability of ending up in low health. We therefore consider individuals with two possible income levels: a ‘low income’ equal to 20,000 AUD (Australian dollar) and a ‘high income’ equal to 80,000 AUD. The low income roughly corresponds to the 10th percentile of the income distribution, whereas the high income roughly corresponds to the 90th percentile. For the other covariates, we set the levels equal to the means in the case of numerical variables and equal to the reference categories in the case of categorical variables. When it comes to smoking, however, we consider two possible categories: we look at the income-health relationship for both non-smokers (the reference category) and smokers. To calculate and compare the GAMLSS results, we follow a similar structure as in [12] and [26].

Table 2 shows the estimated covariate effects on the two parameters (or more precisely, on the logarithms of these parameters) of the gamma distribution, fitted to the transformed health score as defined by Eq. (4). All covariates have a significant impact on *μ* at the 5% level, whereas some covariates are insignificant for predicting *s*. Because most covariates significantly influence both *μ* and *s*, their relationship to the transformed health score goes beyond the mean. The main variable of interest, *log*(income), has a significant negative impact on *log*(*μ*), but does not affect *log*(*s*). The negative relationship with respect to the mean transformed health score confirms previous findings that income has a positive impact on health. As a matter of fact, if we wish to know the effect of the covariates on health, we need to reverse the sign of the coefficients predicting *μ*.

**Table 2:** Linear effects on *log*(*μ*) and *log*(*s*) for the transformed health variable

*Variable*	*log*(*μ*)	*log*(*s*)
*log*(Income)	−0.043^∗∗^	−0.002
Indigenous	0.067^∗∗^	0.019
Age	0.003^∗∗^	−0.005^∗∗^
Age squared	0.000^∗∗^	0.000^∗∗^
Children 0-4	−0.072^∗∗^	0.017
Children 5-14	−0.021^∗∗^	−0.013
Managers & professionals	−0.023^∗^	0.020
Manual workers	0.036^∗^	0.033
Unemployed	0.133^∗∗^	−0.003
Not in labour force	0.183^∗∗^	−0.063^∗∗^
Smoking	0.058^∗∗^	−0.007
Very good sleep quality	−0.154^∗∗^	0.263^∗∗^
Fairly bad sleep quality	0.143^∗∗^	−0.050^∗∗^
Very bad sleep quality	0.233^∗∗^	−0.144^∗∗^
Not reported	0.142^∗∗^	0.028
Almost always stressed	0.150^∗∗^	−0.060^∗∗^
Often stressed	0.089^∗∗^	−0.034^∗^
Rarely stressed	−0.099^∗∗^	0.190^∗∗^
Never stressed	−0.163^∗∗^	0.587^∗∗^
Life satisfaction	−0.069^∗∗^	0.087^∗∗^
Very satisfied with weight	−0.084^∗∗^	0.159^∗∗^
Satisfied with weight	−0.027^∗^	0.033^∗^
Dissatisfied with weight	0.031^∗∗^	−0.100^∗∗^
Very dissatisfied with weight	0.121^∗∗^	−0.135^∗∗^
No physical activity	0.193^∗∗^	−0.183^∗∗^
Some physical activity	0.081^∗∗^	−0.150^∗∗^
Constant	−1.074^∗∗^	−1.097^∗∗^
Global deviance	-26,109.42	
AIC	-26,001.42	
BIC	-25,591.15	

*Note.*^∗^*p*<0.05;^∗∗^*p*<0.01

**Fig. 2:**
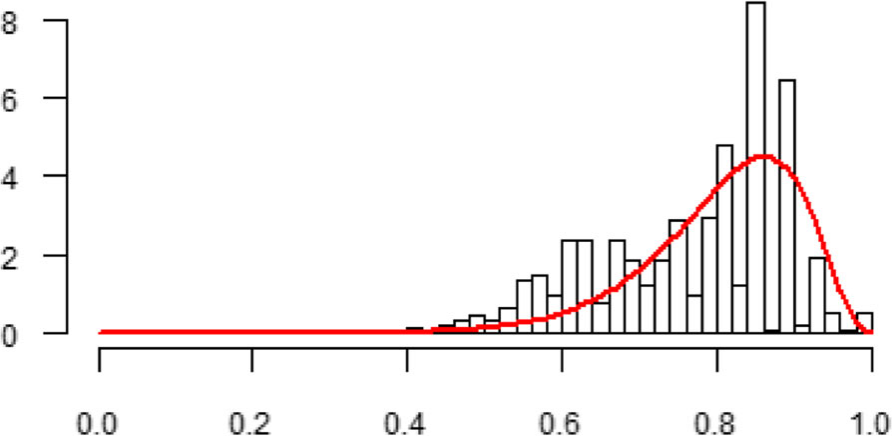
Histogram of the SF-6D health score with fit to the transformed gamma distribution for the reference person

Since the link functions that are used for the predictors are logarithmic instead of linear, covariate effects on the parameters vary across the covariate space. This implies that the impact on *μ* and *s* of a change in income depends on the values of all covariates. To analyse the distributional effect of income on health, we retrieve the parameters of the gamma distribution of the transformed health score for the reference person. That is, we use the means as levels for the numerical variables and the reference categories for the categorical variables. It is worth bearing in mind that the results might be different if we chose another reference person. Figure 2 shows a histogram of the SF-6D health scores together with the transformed gamma distribution when income is set equal to its sample mean.

We analyse the effect on the SF-6D health score when income is changed from 20,000 AUD to 80,000 AUD via five distributional measures. Next to the expectation and standard deviation of the estimated conditional health distribution, we compute three measures that focus on the lower end of the health distribution. We consider three thresholds for health, set at what we consider to be ‘average health’ (*h*≤0.8), ‘fair health’ (*h*≤0.7) and ‘poor health’ (*h*≤0.6). These levels correspond to the 50th, 30th and 10th percentiles of the empirical distribution of the SF-6D health scores. Formally, our measures estimate the risk or probability that a person with income *y* and covariates *X* attains a health outcome below these thresholds:

$$\begin{array}{*{20}l} P_{\text{\scriptsize avg}} = P(h \leq 0.8) \\ P_{\text{\scriptsize fair}} = P(h \leq 0.7) \\ P_{\text{\scriptsize poor}} = P(h \leq 0.6) \end{array} $$

where the health variable *h* follows the conditional distribution for a person with income *y* and covariates *X*.

Table 3 shows the selected health distribution and risk statistics for smokers and non-smokers. For both of these groups, a higher income is associated with a higher expected health level, a lower dispersion of health and smaller probabilities of ending up in bad health. Comparing smokers to non-smokers, we observe that the profile of rich smokers is similar to the one of poor non-smokers. With respect to the risks of ending up in average, fair or poor health, we find that overall the lower the health threshold, the smaller the effect in absolute terms, but the larger in relative terms. The absolute differences appear to be slightly larger for smokers than for non-smokers. The relative differences, however, show an opposite pattern.

**Table 3:** Five measures on the income-health relationship from fitting a gamma distribution to the transformed health variable using GAMLSS regression

	$20,000	$80,000	Absolute difference	Relative difference
a) Smokers				
*E(h)*	0.789 [0.786; 0.797]	0.802 [0.800; 0.809]	0.012 [0.008; 0.017]	1.53% [ 1.06%; 2.05%]
*σ*	0.110 [0.097; 0.115]	0.104 [0.091; 0.107]	0.007 [0.001; 0.013]	6.02% [ 0.85%; 12.14%]
*P*_avg_	0.469 [0.443; 0.485]	0.425 [0.399; 0.436]	0.045 [0.033; 0.062]	9.48% [7.28%; 13.17%]
*P*_fair_	0.188 [0.156; 0.195]	0.154 [0.123; 0.159]	0.033 [0.018; 0.051]	17.73% [10.66%; 27.90%]
*P*_poor_	0.062 [0.042; 0.068]	0.046 [0.029; 0.050]	0.016 [0.006; 0.026]	25.80% [11.71%; 43.44%]
b) Non-smokers				
*E(h)*	0.801 [0.796; 0.806]	0.813 [0.808; 0.817]	0.012 [0.008; 0.016]	1.43% [0.96%; 1.92%]
*σ*	0.105 [0.096; 0.114]	0.099 [0.090; 0.107]	0.006 [0.001; 0.013]	6.02% [0.60%; 11.81%]
*P*_avg_	0.426 [0.406; 0.447]	0.382 [0.364; 0.399]	0.044 [0.030; 0.059]	10.38% [7.31%; 13.55%]
*P*_fair_	0.157 [0.138; 0.175]	0.127 [0.109; 0.143]	0.030 [0.014; 0.047]	19.03% [9.45%; 28.44%]
*P*_poor_	0.048 [0.036; 0.060]	0.035 [0.025; 0.045]	0.013 [0.004; 0.023]	27.38% [8.67%; 43.42%]

*Note.* 95th percentile bootstrap confidence intervals denoted in brackets

**Fig. 3:**
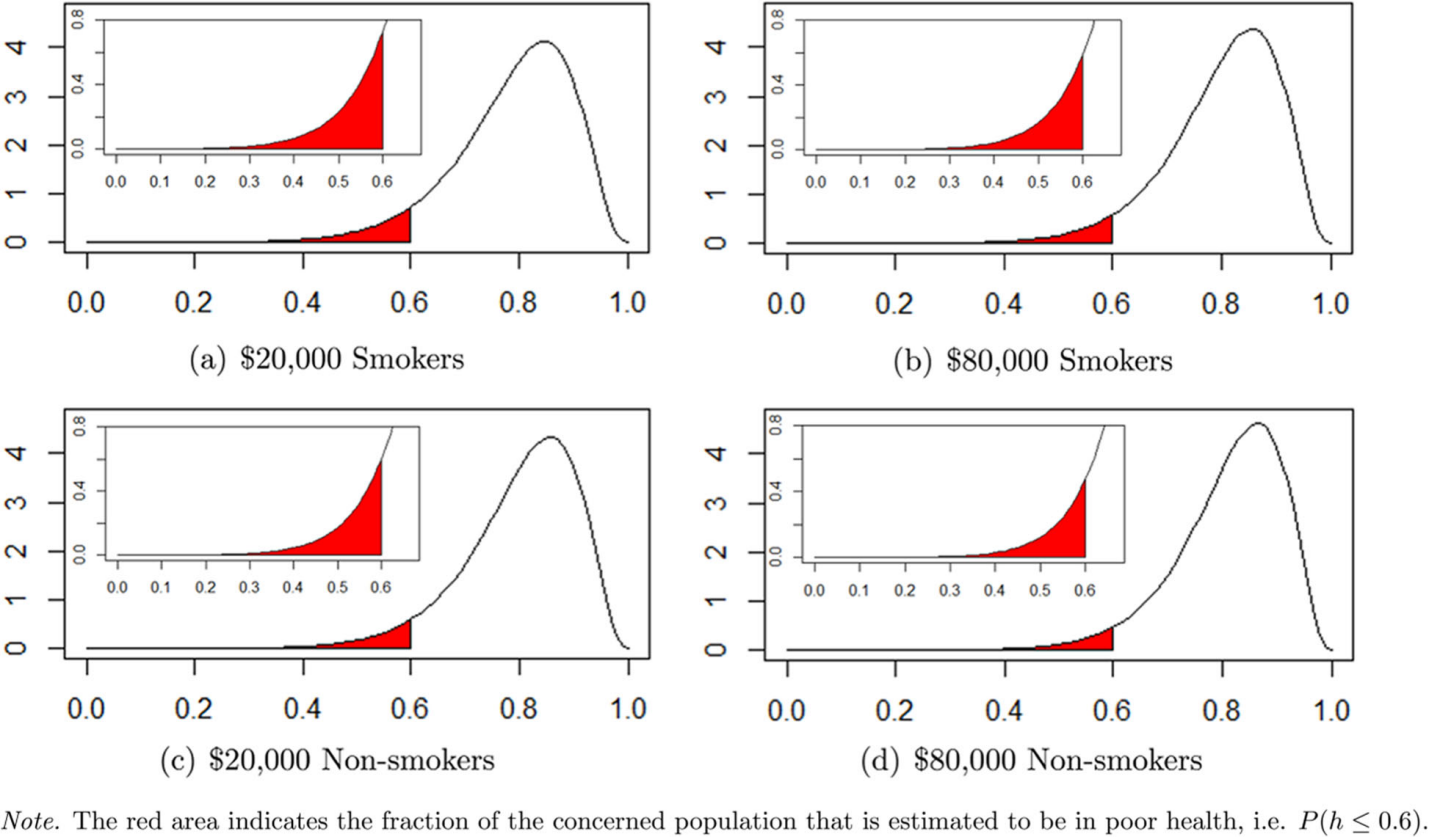
Conditional health distributions obtained by means of GAMLSS regression for smokers (top) and non-smokers (bottom) with incomes of $20,000 (left) and $80,000 (right)

On the whole, the different distributional measures indicate that the income-health relationship is stronger at the lower end of the health distribution. Only looking at the relative difference in expected health scores, i.e. 1.53% for smokers and 1.43% for non-smokers, the effect of income appears to be rather modest. However, when comparing the relative difference in the risk of having average health, i.e. 9.48% for smokers and 10.38% for nonsmokers, to the relative difference in the risk of having poor health, i.e. 25.80% for smokers and 27.38% for non-smokers, we observe that income has a much larger effect at the bottom of the health distribution. Figure 3 illustrates the effect of income on the risk of poor health for both smokers and non-smokers. All of this indicates that the association between income and health goes beyond the mean.

To assess the robustness of our GAMLSS results based on the gamma distribution, we repeated the GAMLSS analysis using two other distributions, the Weibull and lognormal distributions. We present the results for the three distributions in Appendix 1. On the whole, the conclusions on the income-health relationship remain the same across all three distributions. However, there are some slight differences. The Weibull model seems to have a good overall fit with the health scores, while the lognormal model appears to mimic the lower part of the empirical health distribution reasonably well. The gamma model turns out to be the compromise model that closely follows the Weibull model on overall goodness-of-fit and the lognormal model on goodness-of-fit of the lower empirical health scores.

### Quantile regression results

We analyse the relation between the health variable *h* and its covariates in nine quantile regressions for quantiles $0.1, 0.2,\dots,0.9$. We first estimate the coefficients, in particular of income and smoking, for the nine quantiles. Thereafter, we highlight changes in the predicted health levels for the quantiles due to an income increase from 20,000 AUD to 80,000 AUD and due to smoking.

**Fig. 4:**
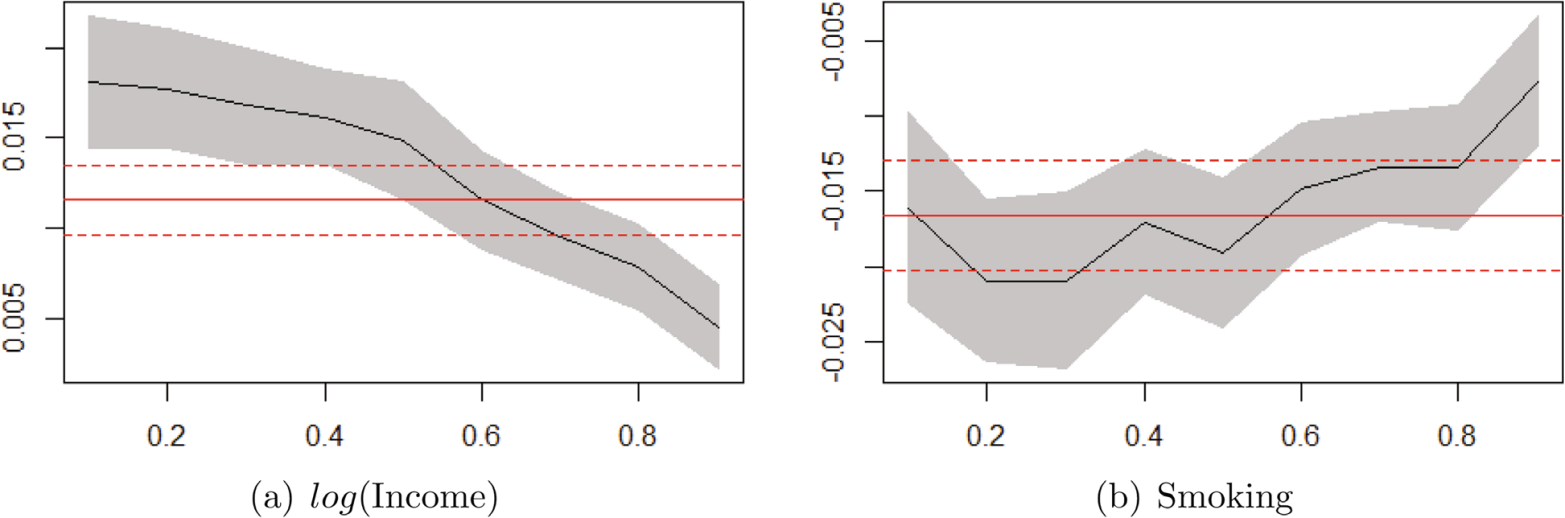
OLS and quantile regression estimates with 95% confidence intervals for the effect of *log*(income) and smoking

Table 10 in Appendix 2 displays the estimated regression coefficients for the nine different quantiles. For comparison, it also contains the effects from OLS (Ordinary Least Squares) regression. We observe that most variables have a significant influence on health in each of the regressions at the 5% level. Moreover, the quantile coefficients vary considerably across different quantiles. For the main covariates of interest, *log*(income) and smoking, the regression coefficients are visualized in Fig. 4a and b.

In both panels a and b, the continuous red line represents the OLS estimate of *log*(income) and smoking, respectively. The dashed red lines represent the 95% confidence interval of the OLS estimate. The nine estimates of the quantile regressions are connected by the black line and the 95% confidence intervals of the quantile regression estimates are shaded grey. Figure 4a shows that the quantile regression coefficients of *log*(income) are larger than the OLS estimate in the lower quantiles of health and smaller in the higher quantiles. This suggests that for the lower tail of the health distribution, OLS regression understates the impact of income on health, whereas for the upper tail of the health distribution, OLS regression overstates the impact of income on health. Similarly, Fig. 4b shows that the quantile regression coefficients of smoking tend to be somewhat larger in absolute magnitude than the OLS estimate in the lower quantiles of health and smaller in the higher quantiles. Changes in income and smoking therefore affect health in a way that is not fully captured by the conditional mean model.

**Fig. 5:**
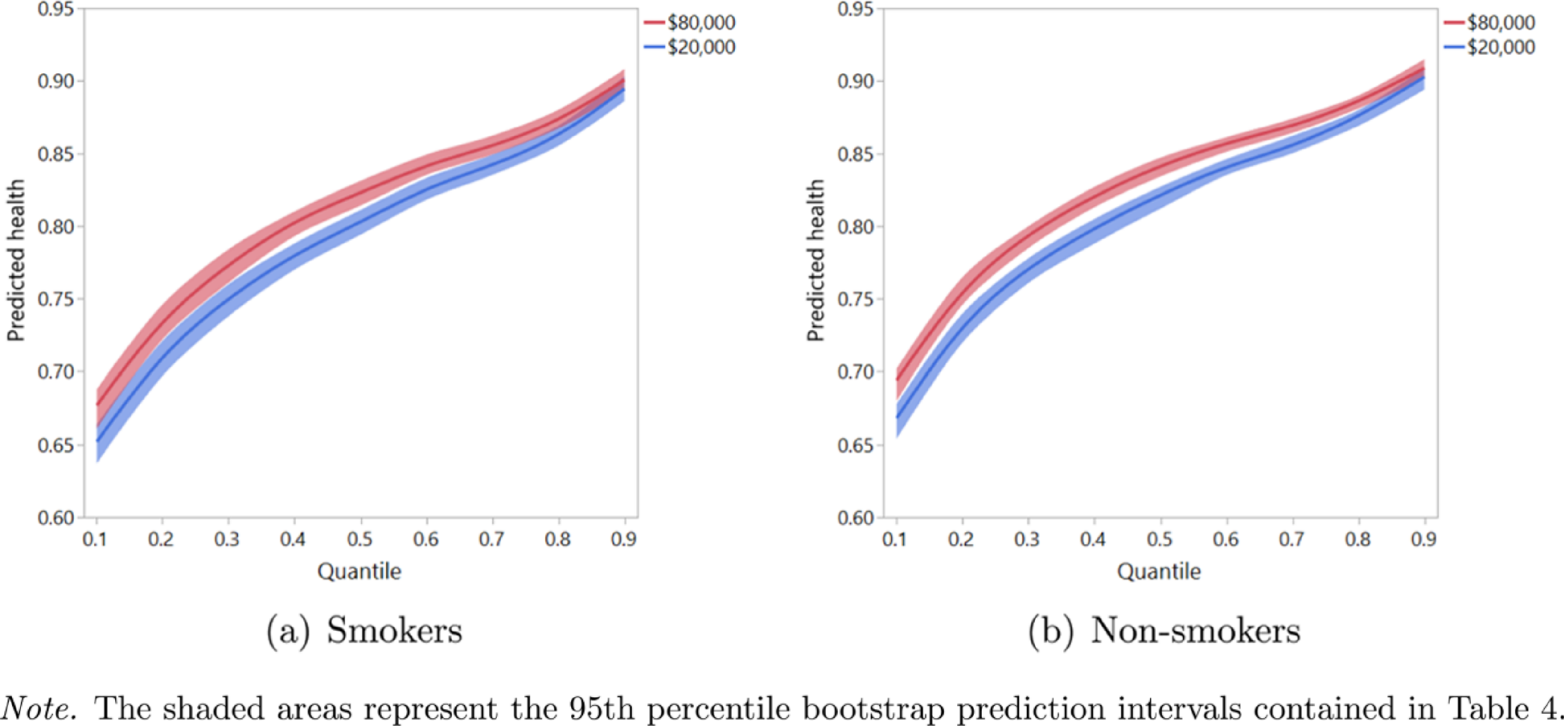
Predicted health quantiles for smokers and non-smokers with incomes of $20,000 and $80,000 and reference values for the other covariates

To illustrate the effect of *log*(income) on health, Table 4 presents the changes in the predicted health quantiles due to an income increase from 20,000 AUD to 80,000 AUD for smokers and non-smokers. Figure 5a and b visualize these values for the two groups. On the whole, people with a high income tend to attain better health outcomes than people with a low income, but the difference in the predicted health values, in both absolute and relative terms, decreases as one’s health improves. Also, comparing smokers to non-smokers, we find that the health profile of rich smokers is fairly similar to that of poor non-smokers, a conclusion we already reached on the basis of the GAMLSS regression results discussed previously.

**Table 4:** Illustration of the effect of *log*(income) on nine predicted health quantiles

*τ*	${\hat {h}^{\tau }(\$20{,}000)}$	${\hat {h}^{\tau }(\$80{,}000)}$	Absolute difference	Relative difference
a) Smokers				
0.1	0.651 [0.637; 0.664]	0.676 [0.661; 0.688]	0.025 [0.017; 0.034]	3.71% [2.47%; 5.04%]
0.2	0.711 [0.697; 0.721]	0.735 [0.722; 0.746]	0.025 [0.020; 0.033]	3.35% [2.70%; 4.46%]
0.3	0.749 [0.738; 0.760]	0.772 [0.761; 0.784]	0.023 [0.018; 0.028]	3.02% [2.28%; 3.64%]
0.4	0.780 [0.770; 0.788]	0.803 [0.793; 0.810]	0.022 [0.018; 0.029]	2.79% [2.21%; 3.58%]
0.5	0.802 [0.794; 0.811]	0.822 [0.814; 0.831]	0.021 [0.015; 0.026]	2.50% [1.78%; 3.15%]
0.6	0.826 [0.818; 0.833]	0.842 [0.835; 0.849]	0.016 [0.012; 0.022]	1.90% [1.42%; 2.59%]
0.7	0.842 [0.835; 0.849]	0.855 [0.849; 0.862]	0.013 [0.009; 0.017]	1.53% [1.06%; 2.01%]
0.8	0.862 [0.855; 0.869]	0.873 [0.867; 0.880]	0.011 [0.007; 0.016]	1.24% [0.82%; 1.84%]
0.9	0.895 [0.886; 0.903]	0.901 [0.893; 0.908]	0.006 [0.002; 0.011]	0.69% [0.18%; 1.26%]
b) Non-smokers				
0.1	0.667 [0.654; 0.678]	0.693 [0.680; 0.702]	0.025 [0.016; 0.035]	3.62% [2.35%; 5.00%]
0.2	0.732 [0.720; 0.740]	0.756 [0.746; 0.765]	0.025 [0.020; 0.034]	3.26% [2.60%; 4.41%]
0.3	0.770 [0.761; 0.778]	0.793 [0.785; 0.800]	0.023 [0.018; 0.028]	2.94% [2.23%; 3.57%]
0.4	0.798 [0.788; 0.805]	0.820 [0.813; 0.827]	0.022 [0.018; 0.029]	2.74% [2.19%; 3.49%]
0.5	0.821 [0.812; 0.827]	0.841 [0.834; 0.847]	0.021 [0.015; 0.026]	2.44% [1.78%; 3.03%]
0.6	0.841 [0.835; 0.846]	0.857 [0.851; 0.861]	0.016 [0.012; 0.021]	1.87% [1.40%; 2.49%]
0.7	0.855 [0.850; 0.862]	0.869 [0.864; 0.874]	0.013 [0.009; 0.017]	1.51% [1.04%; 1.96%]
0.8	0.876 [0.869; 0.880]	0.886 [0.881; 0.890]	0.011 [0.007; 0.016]	1.22% [0.80%; 1.83%]
0.9	0.903 [0.894; 0.909]	0.909 [0.902; 0.915]	0.006 [0.002; 0.011]	0.69% [0.18%; 1.24%]

*Note.* 95th percentile bootstrap prediction intervals denoted in brackets

### Comparison of regression results

We compare the results we previously obtained by means of the GAMLSS technique in two different ways to the quantile regression results, the first of which we call empirical (or non-parametric), and the second parametric. We construct the empirical distribution by estimating a thousand quantile regressions for quantiles $0.001, 0.002,\dots,0.999$ to obtain a better approximation of the distribution of health. This allows us to compute the distributional measures *P*_avg_, *P*_fair_ and *P*_poor_, as defined above, for low and high income earners as well as for smokers and non-smokers. The second method consists of estimating the parameters of a gamma distribution based on the predicted health quantiles instead of the observed health outcomes. Using the gamma distributions for smokers and non-smokers, we calculate the five distributional measures for the income-health relationship.

Table 5 contains the three risk measures from estimating the health distribution using a thousand quantile regressions, without assuming a parametric distribution for the health variable. Overall, we find results that are similar to those from GAMLSS in Table 3. An income increase reduces the probability of ending up in bad health, where the absolute effect is smaller for smaller threshold values for health, and the relative effect larger. Relatively speaking, the income-health relationship is thus stronger at the lower end of the health distribution. This distributional effect is even more pronounced for the quantile regression method than for GAMLSS, because the absolute and relative differences between the risk estimates are generally larger. Comparing smokers to non-smokers, we again notice the similarity in risk profile between a rich smoker and a poor non-smoker. Also, the absolute differences are larger for smokers than for non-smokers. This holds for the relative differences too, whereas we observed the opposite from using GAMLSS.

**Table 5:** Three risk measures on the income-health relationship from using the empirical distribution of 1000 predicted health quantiles

	$20,000	$80,000	Absolute difference	Relative difference
a) Smokers				
*P*_avg_	0.492 [0.454; 0.526]	0.391 [0.362; 0.428]	0.101 [0.065; 0.128]	20.53% [13.76%; 25.15%]
*P*_fair_	0.179 [0.151; 0.204]	0.131 [0.114; 0.154]	0.048 [0.027; 0.067]	26.82% [16.49%; 35.06%]
*P*_poor_	0.041 [0.026; 0.054]	0.025 [0.018; 0.036]	0.016 [0.003; 0.025]	39.02% [10.00%; 52.00%]
b) Non-smokers				
*P*_avg_	0.409 [0.382; 0.445]	0.327 [0.298; 0.354]	0.082 [0.062; 0.115]	20.05% [15.42%; 27.07%]
*P*_fair_	0.143 [0.128; 0.163]	0.114 [0.097; 0.126]	0.029 [0.019; 0.050]	20.28% [14.18%; 32.24%]
*P*_poor_	0.028 [0.019; 0.040]	0.021 [0.015; 0.027]	0.007 [0.000; 0.017]	25.00% [ 0.00%; 46.43%]

*Note.* 95th percentile bootstrap confidence intervals denoted in brackets

**Fig. 6:**
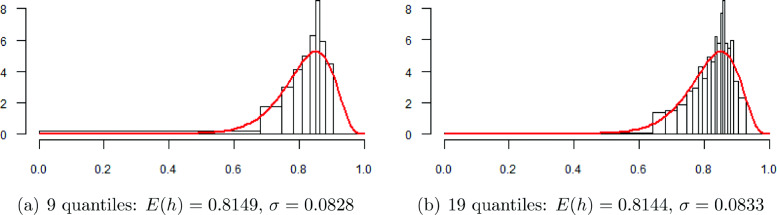
Histograms of 9 and 19 predicted health quantiles with fit to the transformed gamma distributions, described by *E*(*h*) and *σ*, for the reference person

The parametric way to obtain the risk measures from quantile regression is to use the predicted health quantiles for given values of the covariates for the estimation of a gamma distribution. In comparison to the empirical approach, the parametric approach requires much fewer quantile regressions. In general, we found that nine predicted health quantiles are enough to obtain robust estimates of the two parameters of the gamma distribution. Using more predicted health quantiles (e.g., 19 rather than 9) leaves the estimates of the parameters virtually unchanged. As an illustration, Fig. 6a and b contain the histograms of 9 and 19 predicted health quantiles with fit to the transformed gamma distributions for the reference person, the parameters of which are the same up to the third or fourth decimal place.

The distributional measures based on nine predicted health quantiles appear in Table 6, but are representative of many more quantiles. In general, the findings are again similar to the GAMLSS results in Table 3, confirming that low-income earners bear a greater health risk at the bottom of the health distribution. One difference, however, is that the standard deviation of the gamma distribution is smaller when obtained from the predicted health quantiles. Also, the differences between the risk estimates of bad health are very large, even larger than those obtained from the empirical distribution of health regression quantiles in Table 5. Nevertheless, both the absolute and the relative differences show a pattern that is similar to the GAMLSS results.

**Table 6:** Five measures on the income-health relationship from fitting a gamma distribution to the transformed values of nine predicted health quantiles

	$20,000	$80,000	Absolute difference	Relative difference
a) Smokers				
*E(h)*	0.787 [0.779; 0.794]	0.807 [0.800; 0.813]	0.020 [0.016; 0.024]	2.46% [ 1.98%; 3.01%]
*σ*	0.094 [0.087; 0.101]	0.084 [0.078; 0.091]	0.010 [0.006; 0.014]	10.20% [ 6.40%; 14.39%]
*P*_avg_	0.498 [0.467; 0.529]	0.411 [0.380; 0.443]	0.087 [0.068; 0.108]	17.41% [13.80%; 21.59%]
*P*_fair_	0.166 [0.142; 0.193]	0.109 [0.088; 0.134]	0.057 [0.043; 0.072]	34.35% [26.39%; 42.29%]
*P*_poor_	0.041 [0.030; 0.055]	0.021 [0.014; 0.031]	0.020 [0.014; 0.028]	49.24% [36.96%; 60.34%]
b) Non-smokers				
*E(h)*	0.804 [0.797; 0.810]	0.824 [0.818; 0.829]	0.020 [0.016; 0.024]	2.42% [ 1.96%; 2.94%]
*σ*	0.088 [0.082; 0.095]	0.078 [0.073; 0.084]	0.010 [0.006; 0.014]	11.18% [ 6.82%; 15.24%]
*P*_avg_	0.422 [0.397; 0.453]	0.330 [0.305; 0.359]	0.093 [0.074; 0.111]	21.92% [17.62%; 26.11%]
*P*_fair_	0.122 [0.104; 0.145]	0.072 [0.058; 0.091]	0.050 [0.037; 0.063]	40.64% [31.80%; 49.06%]
*P*_poor_	0.026 [0.019; 0.036]	0.011 [0.007; 0.018]	0.015 [0.009; 0.021]	56.11% [42.94%; 66.99%]

*Note.* 95th percentile bootstrap confidence intervals denoted in brackets

## Discussion

The two regression methods explored in this paper - GAMLSS and quantile regression - both allow a more refined analysis of the income-health relationship than conventional regression techniques. GAMLSS makes room for differences in the effects of income on health for different types of individuals, such as low and high income earners, or smokers and non-smokers, using parametric estimates of conditional health distributions. Quantile regression methods do so by estimating the effect of income on health at different locations of the health distribution, and hence go beyond standard regression methods such as OLS, which predict the effect of income on conditional mean health.

Whether one of the two techniques is superior to the other remains an open question, and certainly not one which this paper will settle. Silbersdorff et al. [12] have assessed different arguments in favour of and against GAMLSS and quantile regression, and have a clear preference for the former. They point out that one advantage of GAMLSS over quantile regression is that GAMLSS is suited for both categorical and continuous response variables, while quantile regression cannot be used for ordered categorical responses, which are frequently employed to measure health. Moreover, GAMLSS is appreciated for the fact that it estimates the complete conditional distribution. To obtain a comparable result with quantile regression, many conditional quantile functions have to be estimated. This argument assumes that the response variable can be approximated reasonably well by a probability distribution such as the gamma or lognormal distribution. If no suitable parametric response distribution is available, then the assumption of a parametric distribution is paradoxically one of the main drawbacks of GAMLSS. In quantile regression, such an assumption is not necessary. It deserves to be pointed out, however, that the GAMLSS framework is compatible with a wide range of useful distributions that go far beyond the exponential family of distributions [27]. For that purpose, separate GAMLSS packages in R have been developed (e.g., the gamlss.mx package for fitting finite mixture distributions).

In our study we approximate the health variable by means of the two-parameter gamma distribution in a frequentist estimation framework. Although this distribution is a simple one, Silbersdorff and Schneider [26] showed that differences in the information criteria with more complex three- and four-parameter distributions were only minor. They recommend the use of the two-parameter gamma distribution because it yields risk measures for the assessment of the income-health relationship that are comparable to those of the more complex distributions. Moreover, three- or four-parameter distributions suffer from decreased estimation stability leading to much wider confidence intervals and other statistical deficiencies. However, note that using a Bayesian instead of a frequentist estimation framework of GAMLSS, such as the Structured Additive Distributional Regression technique applied by [12], many of the computational problems can be sidestepped. Under the Bayesian framework, the assumption of a parametric distribution entails estimation stability, especially for samples of limited size and in the tails of the distribution, which are critical for evaluating risks.

Our empirical application has focused on the assessment of the risks of ending up in bad health, using different threshold values for what constitutes bad health (average, fair or poor health). Broadly speaking, we found that GAMLSS and quantile regression gave similar results. Not surprisingly, we consistently observed that low-income earners (i.e., with an equivalent household income of 20,000 AUD) have higher risks than high-income earners (i.e., with an equivalent household income of 80,000 AUD), and that smokers have higher risks than non-smokers. Our results show that the health risk profile of high-income smokers is similar to that of low-income non-smokers, whatever the method we use to calculate the risks. Nevertheless, there are some differences in the risk estimates according to the method adopted. For instance, if we compare the GAMLSS results (Table 3) to those obtained by fitting a gamma distribution to the predicted health quantiles (Table 6), we find that both the absolute and the relative differences between the risk estimates *P*_avg_, *P*_fair_ and *P*_poor_ of the low-income and high-income earners are always larger for the quantile regression approach than for the GAMLSS approach. The absolute and relative differences also tend to be larger if we use the results of the empirical distribution of the predicted health quantiles (Table 5) instead of those of the fitted gamma distribution, but not always. Similar conclusions hold if we compare the risk estimates for smokers and non-smokers. This implies that a comparison of a poor smoker and a rich non-smoker yields larger differences using the quantile regression methods. For instance, the absolute difference in the risks of having average health (*P*_avg_) is equal to 0.469−0.382=0.087 according to the GAMLSS estimates, but equal to 0.492−0.327=0.165 and 0.498−0.330=0.168 according to the two sets of estimates based on quantile regression, i.e. almost twice as large. Although it seems that in this particular case the quantile regression approach is more sensitive to the effects of income and smoking on health than the GAMLSS approach, it remains to be seen whether this holds in general.

Finally, we would like to point out that the assessment of the risks of ending up in bad health is comparable to the measurement of health poverty recently proposed by [36]. The guiding idea is to zoom in on the bottom of the health distribution and to find out if there are groups of the population which are more vulnerable than others. As we have seen, the GAMLSS technique allows us to generate counterfactual probability distributions for specific subgroups, such as rich and poor, controlling for other differences that might exist. We have also indicated how a similar thing can be done by means of quantile regressions, in two different ways. The construction of these distributions requires quite a few assumptions (e.g., about the reference values of the covariates) and a lot of estimates (e.g., quantile regressions). The health poverty approach, by contrast, relies on the subgroup decomposability property of the health poverty indicator and is computationally much simpler. For example, we can divide the population in different income groups and calculate the health poverty index for each income group. However, the drawback is that in this way we cannot control for the effect of other covariates.

## Conclusions

In this paper, we have explored the effect of income on health, using Australian household survey data. In reaction to the limitations of conventional mean-oriented regression techniques, we chose two unconventional regression techniques to study the income-health relationship. Our strategy consisted of using both GAMLSS and quantile regression to estimate conditional health distributions. This allowed us to assess the risks of ending up in bad health for different subgroups of the population. We focused in particular on the differences between low-income and high-income earners, and between smokers and non-smokers. Both regression techniques indicate quite strongly that people with low incomes face higher risks than people with high incomes. But we also found that the magnitude of the difference in risk changes with the chosen threshold for bad health. This suggests that it makes sense to look for regression techniques which are capable of identifying how large the effect of income is on health at different locations of the health distribution. When conventional regression techniques such as OLS find that income has a positive and significant coefficient in the health regression, what it means is that income has a positive effect on mean health. It is impossible to tell from this result whether the effect is smaller or larger at other health levels.

If we are interested in unraveling the causal relationship between income and health in all its complexity, a strong case can therefore be made for the application of distribution-sensitive regression techniques alongside the conventional mean-oriented regression techniques. In addition, the results from a distribution-sensitive regression analysis are helpful when it comes to the formulation of public health policies. The finding that at the lower end of the health spectrum income appears to have a larger effect on health than at the higher end is obviously relevant information for policymakers, especially if they give priority to improving the situation of people in bad health.

Although we compared the results of the GAMLSS technique to those of the quantile regression technique, we refrained from expressing a preference for one or the other. The results of our application of the two techniques are broadly similar, but not identical. However, the scope of our empirical study is too narrow to make general claims about the strengths and weaknesses of both approaches. More research is needed to see how the two compare in different contexts.

Finally, attention should be paid to the limitations of our study. One of the main drawbacks of this research is that it does not take into account the possibility of reverse causality. From the literature we know not only that income tends to have a positive effect on health, but also that health tends to have a positive effect on income. If this is the case, then estimating the impact of income on health by itself creates an endogeneity problem. Possible solutions for this problem are either to study only the impact of truly exogenous income variations on health, or to apply instrumental variable (IV) techniques.

In their appendix, Hohberg, Pütz and Kneib [24] propose an IV method for GAMLSS that is similar to the one Marra and Radice [37] developed for generalized additive models (GAM) which only describe the mean or location of the response distribution. The method exploits the two-stage procedure idea first proposed by Hausman [38, 39] as a means to test for endogeneity. The first stage obtains the residuals from an auxiliary GAM regression of the endogenous variables on all instrumental variables and all exogenous variables. The distributional part comes in the second stage where the residuals from the first stage are added to the GAMLSS model next to the exogenous explanatory variables. In quantile regression, the use of IVs has been pioneered by Chernozhukov and Hansen [40] who derived a set of conditions for identification of the IV quantile regression model without functional form assumptions. Subsequently, Chernozhukov and Hansen [41] proposed the IV quantile regression estimator, which is a quantile analog of two-stage least squares. We refer to [42] for an overview of empirical applications. As is the case with any IV method, the major drawback is the difficulty to select appropriate instruments.

Another limitation of our study is that all distributional results are conditional: with the exception of income and smoking habits, for which we have chosen two possible levels, we assume specific values for all other respondent characteristics to allow for comparisons among distributions. We have found these comparisons to be quite stable because we hardly observed significant effects among the covariates themselves, especially in relation to income. In future research one might consider several types of reference persons by assuming different covariate combinations.

## Appendix 1. GAMLSS results based on the weibull and lognormal distribution

As possible alternatives to the two-parameter gamma distribution in the GAMLSS regression of the SF-6D health score, we chose the two-parameter Weibull and lognormal distributions and computed the associated risks for smokers and non-smokers to experience average, fair or poor health. We specified the Weibull and lognormal distribution by the GAMLSS descriptions WEI3 and LOGNO which refer to the formulations provided by Rigby et al. [27] (see p. 280 for the Weibull distribution and p. 275 for the lognormal distribution).

**Table 7:** Global deviance and information criterion values from using different distributions in GAMLSS regression of the SF-6D health score

Distribution	Global deviance	AIC	BIC
Gamma (GA)	−26,109.42	−26,001.42	−25,591.15
Weibull (WEI3)	−27,025.01	−26,917.01	−26,506.74
Lognormal (LOGNO)	−19,339.60	−19,231.60	−18,821.33

To compare the performance of the Weibull and lognormal distribution to that of the gamma distribution, we first study the fit of these distributions in the GAMLSS models by means of the global deviance and AIC and BIC information criteria. Table 7 compares the values for the different distributions and shows that the smallest values are obtained with the Weibull distribution, although they come close to those of the gamma distribution. The lognormal distribution has the highest values indicating lower goodness-of-fit.

**Fig. 7:**
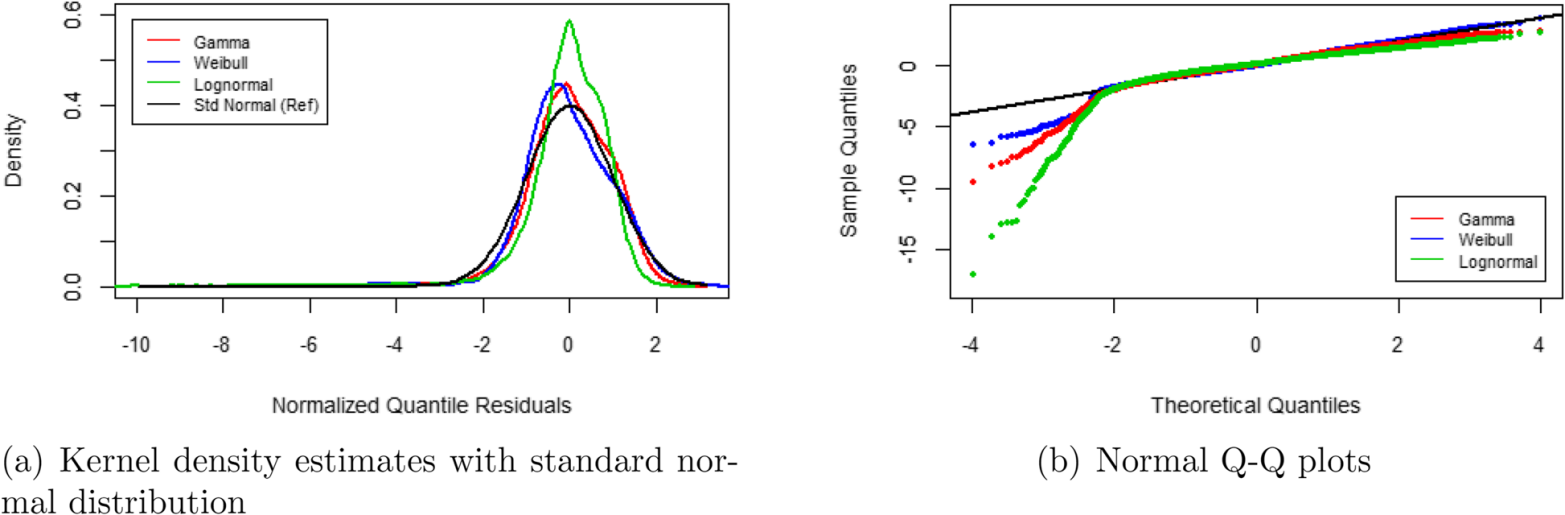
Diagnostic plots of normalized quantile residuals from GAMLSS regressions of the SF-6D health score, based on the transformed gamma, Weibull and lognormal distribution

**Fig. 8:**
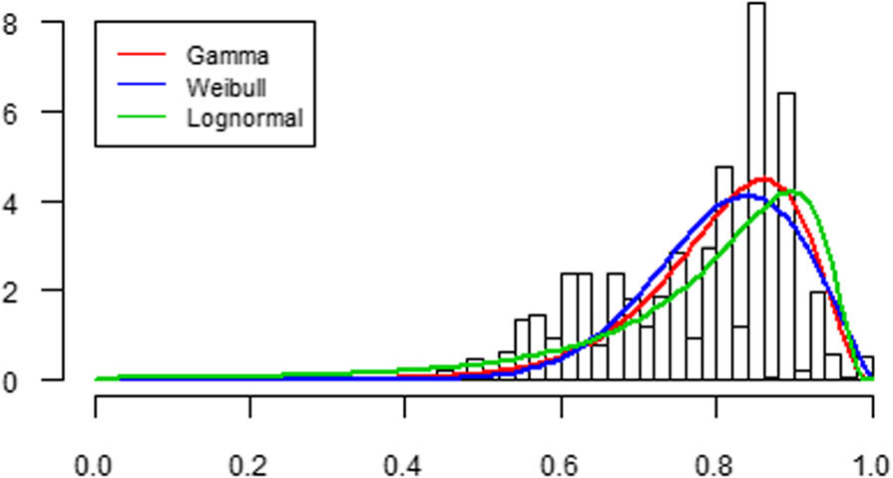
Histogram of the SF-6D health score with fit to the transformed gamma, Weibull and lognormal distribution for the reference person

Figure 7a and b show diagnostic plots of the normalized (randomized) quantile residuals for the GAMLSS models to further evaluate the adequacy of the model distributions. We use the normalized quantile residuals because they follow a standard normal distribution when the assumed model is correct, similar to traditional residuals from linear models [43]. Panel a presents the kernel density estimates of the residuals from the GAMLSS models as well as the standard normal distribution for comparison. Panel b shows the corresponding Quantile-Quantile plots. Both panels reveal that the residuals of the lognormal model deviate the most from the standard normal distribution, and those of the Weibull and gamma models the least. All three distributions are negatively skewed and leptokurtic, where the lognormal distribution has a skewness (-3.76) and kurtosis (36.53) that are far from optimal (i.e., compared to a skewness of 0 and kurtosis of 3 for the standard normal distribution). The lognormal model seems therefore less appropriate.

Figure 8 plots the estimated GAMLSS distributions for the reference person (non-smoker) on the histogram of the SF-6D health score. Overall, both the gamma and Weibull distribution seem to summarize the data better than the lognormal distribution. When focusing on the lower part of the histogram, however, the lognormal distribution appears to describe the data better by its fatter tail compared to the gamma and Weibull distribution.

Tables 8 and 9 show the summary and risk statistics for smokers and non-smokers based on the Weibull and lognormal distribution. In general, the trends we observe are similar to those for the gamma distribution in Table 3. However, the results in the tables differ in the ability of the distributions to describe the empirical health scores at the lower end. The lognormal distribution captures the lowest health scores best. That is to say, the risk probabilities *P*_fair_ and *P*_poor_ from the lognormal distribution come closest to the empirical cumulative probabilities of 30% and 10% for the non-smokers and 40% and 20% for the smokers, respectively. On the other hand, the Weibull distribution describes the lowest health scores worst.

To conclude, the gamma model turns out to be the compromise between the Weibull model that performs best on overall goodness-of-fit and the lognormal model that performs best on fitting the lower part of the empirical health distribution. The gamma model has a reasonable overall goodness-of-fit that is comparable to that of the Weibull model and an ability to capture the lower empirical health scores to some extent, but not as well as the lognormal model.

**Table 8:** Five measures on the income-health relationship from fitting a Weibull distribution to the transformed health variable using GAMLSS regression

	$20,000	$80,000	Absolute difference	Relative difference
a) Smokers				
*E(h)*	0.791 [0.786; 0.797]	0.803 [0.798; 0.808]	0.012 [0.008; 0.015]	1.44% [ 1.04%; 1.87%]
*σ*	0.099 [0.093; 0.103]	0.094 [0.089; 0.097]	0.005 [0.001; 0.008]	4.63% [ 1.50%; 8.03%]
*P*_avg_	0.499 [0.479; 0.525]	0.453 [0.433; 0.475]	0.046 [0.034; 0.060]	9.20% [ 6.97%; 11.83%]
*P*_fair_	0.180 [0.159; 0.194]	0.145 [0.126; 0.156]	0.035 [0.024; 0.049]	19.66% [13.46%; 26.74%]
*P*_poor_	0.039 [0.027; 0.046]	0.026 [0.018; 0.031]	0.012 [0.006; 0.019]	32.30% [18.93%; 46.65%]
b) Non-smokers				
*E(h)*	0.802 [0.797; 0.807]	0.813 [0.809; 0.818]	0.011 [0.008; 0.014]	1.35% [ 0.97%; 1.75%]
*σ*	0.097 [0.093; 0.102]	0.093 [0.088; 0.097]	0.004 [0.001; 0.008]	4.62% [ 1.49%; 8.04%]
*P*_avg_	0.453 [0.433; 0.476]	0.408 [0.390; 0.428]	0.045 [0.033; 0.058]	9.89% [ 7.37%; 12.58%]
*P*_fair_	0.152 [0.135; 0.168]	0.121 [0.107; 0.135]	0.031 [0.020; 0.043]	20.38% [13.37%; 27.40%]
*P*_poor_	0.031 [0.023; 0.039]	0.021 [0.015; 0.027]	0.010 [0.005; 0.016]	32.66% [17.85%; 46.48%]

*Note.* 95th percentile bootstrap confidence intervals denoted in brackets

**Table 9:** Five measures on the income-health relationship from fitting a lognormal distribution to the transformed health variable using GAMLSS regression

	$20,000	$80,000	Absolute difference	Relative difference
a) Smokers				
*E(h)*	0.759 [0.733; 0.773]	0.770 [0.742; 0.784]	0.011 [0.001; 0.031]	1.41% [ 0.08%; 3.99%]
*σ*	0.194 [0.148; 0.266]	0.188 [0.143; 0.263]	0.006 [0.001; 0.059]	3.19% [ 0.34%; 24.25%]
*P*_avg_	0.465 [0.452; 0.489]	0.436 [0.426; 0.458]	0.028 [0.012; 0.046]	6.06% [ 2.57%; 9.66%]
*P*_fair_	0.254 [0.223; 0.290]	0.233 [0.200; 0.274]	0.020 [0.001; 0.056]	8.03% [ 0.33%; 20.26%]
*P*_poor_	0.142 [0.107; 0.183]	0.129 [0.093; 0.173]	0.013 [0.001; 0.049]	9.29% [ 0.47%; 29.94%]
b) Non-smokers				
*E(h)*	0.774 [0.756; 0.790]	0.784 [0.764; 0.801]	0.010 [0.001; 0.028]	1.30% [ 0.07%; 3.47%]
*σ*	0.182 [0.133; 0.236]	0.176 [0.129; 0.233]	0.006 [0.000; 0.051]	3.20% [ 0.30%; 23.91%]
*P*_avg_	0.429 [0.408; 0.447]	0.401 [0.382; 0.419]	0.027 [0.009; 0.050]	6.39% [ 2.03%; 11.47%]
*P*_fair_	0.226 [0.184; 0.256]	0.207 [0.162; 0.242]	0.019 [0.001; 0.055]	8.34% [ 0.25%; 23.44%]
*P*_poor_	0.123 [0.082; 0.155]	0.111 [0.070; 0.146]	0.012 [0.001; 0.045]	9.57% [ 0.52%; 33.59%]

*Note.* 95th percentile bootstrap confidence intervals denoted in brackets

## Appendix 2. OLS and quantile regressions of health

**Table 10:** OLS and quantile regression estimates for quantiles $\tau _{1} = 0.1, \dots, \tau _{9} = 0.9$ of the health variable

Variable	OLS	*τ*_1_	*τ*_2_	*τ*_3_	*τ*_4_	*τ*_5_	*τ*_6_	*τ*_7_	*τ*_8_	*τ*_9_
*log*(Income)	$\underset {(0.001)}{\hspace {2.3pt}0.012}^{**}$	$\underset {(0.002)}{\hspace {2pt} 0.018}^{**}$	$\underset {(0.002)}{\hspace {3pt} 0.018}^{**}$	$\underset {(0.002)}{\hspace {1.7pt}0.017}^{**}$	$\underset {(0.002)}{\hspace {2pt} 0.016}^{**}$	$\underset {(0.002)}{\hspace {1.9pt}0.015}^{**}$	$\underset {(0.002)}{\hspace {2.5pt}0.012}^{**}$	$\underset {(0.001)}{\hspace {1.8pt}0.009}^{**}$	$\underset {(0.001)}{\hspace {2.5pt}0.008}^{**}$	$\underset {(0.001)}{\hspace {2pt} 0.005}^{**}$
Indigenous	$\underset {\phantom {-}(0.006)}{\hspace {1pt} -0.020}^{**}$	$\underset {\phantom {-}(0.011)}{\hspace {1pt} -0.029}^{**}$	$\underset {\phantom {-}(0.009)}{-0.027}^{**}$	$\underset {\phantom {-}(0.008)}{-0.026}^{**}$	$\underset {\phantom {-}(0.009)}{\hspace {1pt} -0.025}^{**}$	$\underset {\phantom {-}(0.008)}{-0.022}^{**}$	$\underset {\phantom {-}(0.008)}{\hspace {1pt} -0.018}^{*\phantom {*}}$	$\underset {\phantom {-}(0.006)}{-0.013}^{*\phantom {*}}$	$\underset {\phantom {-}(0.006)\phantom {^{**}}} {\hspace {2pt} -0.004\phantom {^{**}}}$	$\underset {\phantom {-}(0.005)\phantom {^{**}}} {\hspace {2.5pt}-0.007\phantom {^{**}}}$
Age	$\underset {\phantom {-}(0.000)}{\hspace {1pt} -0.001 }^{**}$	$\underset {\phantom {-}(0.000)}{\hspace {1pt} -0.001}^{**}$	$\underset {\phantom {-}(0.000)}{-0.001 }^{**}$	$\underset {\phantom {-}(0.000)}{\hspace {.5pt}-0.001 }^{**}$	$\underset {\phantom {-}(0.000)}{-0.001 }^{**}$	$\underset {\phantom {-}(0.000)}{-0.001 }^{**}$	$\underset {\phantom {-}(0.000)}{\hspace {1pt} -0.001 }^{**}$	$\underset {\phantom {-}(0.000)}{-0.001 }^{**}$	$\underset {\phantom {-}(0.000)}{-0.001 }^{**}$	$\underset {\phantom {-}(0.000)}{-0.001 }^{**}$
Age squared	$\underset {(0.000)}{\hspace {2.4pt}0.000}^{**}$	$\underset {(0.000)}{\hspace {2pt} 0.000}^{**}$	$\underset {(0.000)}{\hspace {2.5pt}0.000}^{**}$	$\underset {(0.000)}{\hspace {2.5pt}0.000}^{**}$	$\underset {(0.000)}{\hspace {2pt} 0.000}^{**}$	$\underset {(0.000)}{\hspace {2pt} 0.000}^{**}$	$\underset {(0.000)}{\hspace {2.5pt}0.000}^{**}$	$\underset {(0.000)}{\hspace {2pt} 0.000}^{**}$	$\underset {(0.000)}{\hspace {2pt} 0.000 }^{**}$	$\underset {(0.000)}{\hspace {2.5pt}0.000}^{**}$
Children 0-4	$\underset {(0.002)}{\hspace {2.5pt}0.017}^{**}$	$\underset {(0.003)} {\hspace {2pt} 0.025}^{**}$	$\underset {(0.002)}{\hspace {2.5pt}0.026}^{**}$	$\underset {(0.003)}{\hspace {2.5pt}0.020}^{**}$	$\underset {(0.002)}{\hspace {2pt} 0.017}^{**}$	$\underset {(0.002)}{\hspace {2pt} 0.016 }^{**}$	$\underset {(0.002)}{\hspace {2.5pt}0.014}^{**}$	$\underset {(0.002)}{\hspace {2pt} 0.013}^{**}$	$\underset {(0.002)}{\hspace {2pt} 0.013}^{**}$	$\underset {(0.001)}{\hspace {2.5pt}0.010}^{**}$
Children 5-14	$\underset {(0.001)}{\hspace {2.2pt}0.005}^{**}$	$\underset {(0.002)}{\hspace {2pt} 0.006}^{*\phantom {*}}$	$\underset {(0.002)}{\hspace {2.5pt}0.007}^{**}$	$\underset {(0.002)}{\hspace {2.5pt}0.007}^{**}$	$\underset {(0.001)}{\hspace {2pt} 0.006}^{**}$	$\underset {(0.001)}{\hspace {2pt} 0.005}^{**}$	$\underset {(0.002)}{\hspace {2.5pt}0.005}^{**}$	$\underset {(0.001)}{\hspace {2pt} 0.006}^{**}$	$\underset {(0.001)}{\hspace {2pt} 0.005}^{**}$	$\underset {(0.001)}{\hspace {2.5pt}0.003}^{*\phantom {*}}$
Managers & professionals	$\underset {\hspace {-3.5pt}(0.002)\phantom {^{**}}}{0.004\phantom {^{**}}}$	$\underset {(0.004)\phantom {^{**}}}{\hspace {3.5pt}0.004\phantom {^{**}}}$	$\underset {\hspace {-3.5pt}(0.004)\phantom {^{**}}}{0.002\phantom {^{**}}}$	$\underset {(0.003)\phantom {^{**}}} {\hspace {2.5pt}0.005\phantom {^{**}}}$	$\underset {(0.003)\phantom {^{**}}}{\hspace {3pt} 0.004\phantom {^{**}}}$	$\underset {(0.002)\phantom {^{**}}}{\hspace {3pt} 0.003\phantom {^{**}}}$	$\underset {(0.002)\phantom {^{**}}}{\hspace {3.5pt}0.002\phantom {^{**}}}$	$\underset {(0.002)\phantom {^{**}}}{\hspace {2.5pt}0.004\phantom {^{**}}}$	$\underset {(0.002)}{\hspace {2pt} 0.004}^{*\phantom {^{*}}}$	$\underset {(0.002)\phantom {^{**}}}{\hspace {2.5pt}0.001\phantom {^{**}}}$
Manual workers	$\underset {\phantom {-}(0.003)}{-0.008}^{**}$	$\underset {\phantom {-}(0.005)}{-0.022}^{**}$	$\underset {\phantom {-}(0.005)}{-0.017}^{**}$	$\underset {\phantom {-}(0.005)}{-0.014}^{**}$	$\underset {\phantom {-}(0.004)} {-0.009}^{*\phantom {*}}$	$\underset {\phantom {-}(0.003)}{-0.005}^{*\phantom {*}}$	$\underset {\phantom {-}(0.003)}{-0.006}^{*\phantom {*}}$	$\underset {\phantom {-}(0.002)}{-0.005}^{*\phantom {*}}$	$\underset {\phantom {-}(0.003)\phantom {^{**}}}{\hspace {2pt} -0.003\phantom {^{**}}}$	$\underset {\phantom {-}(0.003)\phantom {^{**}}}{\hspace {2.5pt}-0.003\phantom {^{**}}}$
Unemployed	$\underset {\phantom {-}(0.005)}{-0.032}^{**}$	$\underset {\phantom {-}(0.010)}{-0.043}^{**}$	$\underset {\phantom {-}(0.008)}{-0.039}^{**}$	$\underset {\phantom {-}(0.006)}{-0.039}^{**}$	$\underset {\phantom {-}(0.006)}{-0.038}^{**}$	$\underset {\phantom {-}(0.006)}{-0.031}^{**}$	$\underset {\phantom {-}(0.005)}{-0.029}^{**}$	$\underset {\phantom {-}(0.005)}{-0.023}^{**}$	$\underset {\phantom {-}(0.007)}{-0.015}^{*\phantom {*}}$	$\underset {\phantom {-}(0.005)}{-0.014}^{**}$
Not in labour force	$\underset {\phantom {-}(0.003)}{-0.047}^{**}$	$\underset {\phantom {-}(0.004)}{-0.059}^{**}$	$\underset {\phantom {-}(0.004)}{-0.060}^{**}$	$\underset {\phantom {-}(0.004)}{-0.061}^{**}$	$\underset {\phantom {-}(0.004)} {-0.055}^{**}$	$\underset {\phantom {-}(0.003)}{-0.045}^{**}$	$\underset {\phantom {-}(0.003)}{-0.040}^{**}$	$\underset {\phantom {-}(0.003)}{-0.032}^{**}$	$\underset {\phantom {-}(0.002)}{-0.027}^{**}$	$\underset {\phantom {-}(0.002)} {-0.023}^{**}$
Smoking	$\underset {\phantom {-}(0.002)}{-0.017}^{**}$	$\underset {\phantom {-}(0.004)}{-0.016}^{**}$	$\underset {\phantom {-}(0.003)}{-0.021}^{**}$	$\underset {\phantom {-}(0.004)}{\hspace {.5pt}-0.021}^{**}$	$\underset {\phantom {-}(0.003)}{-0.017}^{**}$	$\underset {\phantom {-}(0.003)}{-0.019}^{**}$	$\underset {\phantom {-}(0.003)}{-0.015}^{**}$	$\underset {\phantom {-}(0.002)}{-0.013}^{**}$	$\underset {\phantom {-}(0.002)}{-0.013}^{**}$	$\underset {\phantom {-}(0.003)}{-0.008}^{**}$
Very good sleep quality	$\underset {(0.002)}{\hspace {2.5pt}0.028}^{**}$	$\underset {(0.004)}{\hspace {2pt} 0.040}^{**}$	$\underset {(0.004)}{\hspace {2.5pt}0.038}^{**}$	$\underset {(0.003)}{\hspace {2.5pt}0.034}^{**}$	$\underset {(0.003)}{\hspace {2pt} 0.029}^{**}$	$\underset {(0.002)}{\hspace {2pt} 0.024}^{**}$	$\underset {(0.002)}{\hspace {2.5pt}0.021}^{**}$	$\underset {(0.002)}{\hspace {2pt} 0.021}^{**}$	$\underset {(0.002)}{\hspace {2pt} 0.020}^{**}$	$\underset {(0.002)}{\hspace {2pt} 0.020}^{**}$
Fairly bad sleep quality	$\underset {\phantom {-}(0.002)}{\hspace {1pt} -0.039 }^{**}$	$\underset {\phantom {-}(0.004)}{-0.040}^{**}$	$\underset {\phantom {-}(0.003)}{-0.049 }^{**}$	$\underset {\phantom {-}(0.003)}{\hspace {.5pt}-0.052}^{**}$	$\underset {\phantom {-}(0.003)}{-0.051}^{**}$	$\underset {\phantom {-}(0.003)} { -0.046 }^{**}$	$\underset {\phantom {-}(0.003)}{-0.041}^{**}$	$\underset {\phantom {-}(0.003)}{-0.032 }^{**}$	$\underset {\phantom {-}(0.003)}{-0.026}^{**}$	$\underset {\phantom {-}(0.003)}{-0.023}^{**}$
Very bad sleep quality	$\underset {\phantom {-}(0.005)}{\hspace {1pt} -0.084 }^{**}$	$\underset {\phantom {-}(0.009)}{-0.092}^{**}$	$\underset {\phantom {-}(0.006)}{-0.089 }^{**}$	$\underset {\phantom {-}(0.007)}{-0.082}^{**}$	$\underset {\phantom {-}(0.005)}{-0.092}^{**}$	$\underset {\phantom {-}(0.005)}{-0.096 }^{**}$	$\underset {\phantom {-}(0.005)}{-0.102}^{**}$	$\underset {\phantom {-}(0.007)}{-0.102 }^{**}$	$\underset {\phantom {-}(0.010)}{-0.068}^{**}$	$\underset {\phantom {-}(0.006)}{-0.052}^{**}$
Not reported	$\underset {\phantom {-}(0.009)}{-0.046}^{**}$	$\underset {\phantom {-}(0.003)}{-0.076}^{**}$	$\underset {\phantom {-}(0.017)}{-0.079 }^{**}$	$\underset {\phantom {-}(0.017)}{-0.080 }^{**}$	$\underset {\phantom {-}(0.018)}{-0.055}^{**}$	$\underset {\phantom {-}(0.018)}{-0.047}^{*\phantom {*}}$	$\underset {\phantom {-}(0.009)}{-0.029 }^{**}$	$\underset {\phantom {-}(0.009)}{-0.026 }^{**}$	$\underset {\phantom {-}(0.010)}{-0.021}^{*\phantom {*}}$	$\underset {\phantom {-}(0.005)}{-0.014}^{**}$
Almost always stressed	$\underset {\phantom {-}(0.003)}{\hspace {1pt} -0.036 }^{**}$	$\underset {\phantom {-}(0.005)}{-0.045}^{**}$	$\underset {\phantom {-}(0.005)}{-0.042}^{**}$	$\underset {\phantom {-}(0.005)}{-0.042}^{**}$	$\underset {\phantom {-}(0.004)}{-0.041}^{**}$	$\underset {\phantom {-}(0.004)}{-0.038 }^{**}$	$\underset {\phantom {-}(0.004)}{-0.034}^{**}$	$\underset {\phantom {-}(0.004)}{-0.029}^{**}$	$\underset {\phantom {-}(0.004)}{-0.028 }^{**}$	$\underset {\phantom {-}(0.003)}{-0.023}^{**}$
Often stressed	$\underset {\phantom {-}(0.002)}{\hspace {1pt} -0.020 }^{**}$	$\underset {\phantom {-}(0.003)}{-0.027 }^{**}$	$\underset {\phantom {-}(0.003)}{-0.026}^{**}$	$\underset {\phantom {-}(0.003)}{-0.027}^{**}$	$\underset {\phantom {-}(0.003)}{-0.025}^{**}$	$\underset {\phantom {-}(0.003)}{-0.020}^{**}$	$\underset {\phantom {-}(0.002)}{-0.017 }^{**}$	$\underset {\phantom {-}(0.002)}{-0.014 }^{**}$	$\underset {\phantom {-}(0.002)}{-0.014 }^{**}$	$\underset {\phantom {-}(0.002)}{-0.012}^{**}$
Rarely stressed	$\underset {(0.002)}{\hspace {2.5pt}0.024}^{**}$	$\underset {(0.004)}{\hspace {2pt} 0.018}^{**}$	$\underset {(0.004)}{\hspace {2.5pt}0.024}^{**}$	$\underset {(0.003)}{\hspace {2.5pt}0.025}^{**}$	$\underset {(0.003)}{\hspace {2.5pt}0.027}^{**}$	$\underset {(0.003)}{\hspace {2pt} 0.027}^{**}$	$\underset {(0.002)}{\hspace {2.5pt}0.026}^{**}$	$\underset {(0.002)}{\hspace {2pt} 0.024}^{**}$	$\underset {(0.002)}{\hspace {2pt} 0.021}^{**}$	$\underset {(0.002)}{0.019}^{**}$
Never stressed	$\underset {(0.006)}{\hspace {2.5pt}0.043}^{**}$	$\underset {(0.010)}{\hspace {2pt} 0.037}^{**}$	$\underset {(0.009)}{\hspace {2.5pt}0.037}^{**}$	$\underset {(0.007)}{\hspace {2.5pt}0.038}^{**}$	$\underset {(0.008)}{\hspace {2pt} 0.034}^{**}$	$\underset {(0.009)}{\hspace {2pt} 0.046}^{**}$	$\underset {(0.005)}{\hspace {2.5pt}0.053}^{**}$	$\underset {(0.005)}{\hspace {2.5pt}0.053}^{**}$	$\underset {(0.008)}{\hspace {2pt} 0.056}^{**}$	$\underset {(0.008)}{\hspace {2pt} 0.058}^{**}$
Life satisfaction	$\underset {(0.001)}{\hspace {2.5pt}0.022}^{**}$	$\underset {(0.001)}{\hspace {2pt} 0.020}^{**}$	$\underset {(0.001)}{\hspace {2.5pt}0.024}^{**}$	$\underset {(0.001)}{\hspace {2.5pt}0.023}^{**}$	$\underset {(0.001)}{\hspace {2pt} 0.023}^{**}$	$\underset {(0.001)}{\hspace {2pt} 0.023}^{**}$	$\underset {(0.001)}{\hspace {2.5pt}0.023}^{**}$	$\underset {(0.001)}{\hspace {2pt} 0.022}^{**}$	$\underset {(0.001)}{\hspace {2pt} 0.019}^{**}$	$\underset {(0.001)}{\hspace {2pt} 0.016}^{**}$
Very satisfied with weight	$\underset {(0.003)}{\hspace {2.5pt}0.016}^{**}$	$\underset {(0.006)}{\hspace {2pt} 0.019}^{**}$	$\underset {(0.006)}{\hspace {2.5pt}0.017}^{**}$	$\underset {(0.004)}{\hspace {2.5pt}0.018}^{**}$	$\underset {(0.004)}{\hspace {2pt} 0.014}^{**}$	$\underset {(0.003)}{\hspace {2pt} 0.012}^{**}$	$\underset {(0.003)}{\hspace {2.5pt}0.013}^{**}$	$\underset {(0.003)}{\hspace {2pt} 0.014}^{**}$	$\underset {(0.002)}{\hspace {2pt} 0.012}^{**}$	$\underset {(0.002)}{\hspace {2pt} 0.016}^{**}$
Satisfied with weight	$\underset {(0.002)}{\hspace {2.5pt}0.006}^{*\phantom {*}}$	$\underset {(0.004)\phantom {^{**}}}{\hspace {3pt} 0.006\phantom {^{**}}}$	$\underset {(0.004)\phantom {^{**}}}{\hspace {2.5pt}0.002\phantom {^{**}}}$	$\underset {(0.003)\phantom {^{**}}}{\hspace {3.5pt}0.005\phantom {^{**}} }$	$\underset {(0.003)\phantom {^{**}}} {\hspace {3pt} 0.005\phantom {^{**}}}$	$\underset {(0.003)\phantom {^{**}}}{\hspace {3pt} 0.005\phantom {^{**}}}$	$\underset {(0.002)}{\hspace {2.5pt}0.005 }^{*\phantom {*}}$	$\underset {(0.002)}{\hspace {2.5pt}0.006}^{**}$	$\underset {(0.002)}{0.005}^{*\phantom {*}}$	$\underset {(0.002)} {0.005}^{*\phantom {*}}$
Dissatisfied with weight	$\underset {\phantom {-}(0.002)}{\hspace {1pt} -0.007}^{**}$	$\underset {\phantom {-}(0.004)\phantom {^{**}}}{\hspace {2pt} -0.005\phantom {^{**}}}$	$\underset {\phantom {-}(0.004)}{-0.010 }^{**}$	$\underset {\phantom {-}(0.003)}{-0.012 }^{**}$	$\underset {\phantom {-}(0.003)}{-0.010 }^{**}$	$\underset {\phantom {-}(0.003)}{-0.011 }^{**}$	$\underset {\phantom {-}(0.002)}{\hspace {1pt} -0.008 }^{**}$	$\underset {\phantom {-}(0.002)}{-0.005 }^{*\phantom {*}}$	$\underset {\phantom {-}(0.002)}{-0.006 }^{**}$	$\underset {\phantom {-}(0.002)}{-0.009}^{**}$
Very dissatisfied with weight	$\underset {\phantom {-}(0.003)}{\hspace {1pt} -0.036}^{**}$	$\underset {\phantom {-}(0.005)}{-0.038 }^{**}$	$\underset {\phantom {-}(0.005)}{-0.043 }^{**}$	$\underset {\phantom {-}(0.005)}{-0.045 }^{**}$	$\underset {\phantom {-}(0.004)}{-0.045}^{**}$	$\underset {\phantom {-}(0.005)}{-0.045}^{**}$	$\underset {\phantom {-}(0.004)}{-0.036}^{**}$	$\underset {\phantom {-}(0.004)}{-0.032}^{**}$	$\underset {\phantom {-}(0.005)}{-0.027 }^{**}$	$\underset {\phantom {-}(0.004)}{-0.026 }^{**}$
No physical activity	$\underset {\phantom {-}(0.003)}{\hspace {1pt} -0.059}^{**}$	$\underset {\phantom {-}(0.005)}{-0.062 }^{**}$	$\underset {\phantom {-}(0.004)} {-0.066}^{**}$	$\underset {\phantom {-}(0.004)}{-0.067}^{**}$	$\underset {\phantom {-}(0.004)} {-0.069}^{**}$	$\underset {\phantom {-}(0.004)}{-0.069}^{**}$	$\underset {\phantom {-}(0.005)}{-0.061}^{**}$	$\underset {\phantom {-}(0.004)}{-0.050}^{**}$	$\underset {\phantom {-}(0.004)}{-0.042}^{**}$	$\underset {\phantom {-}(0.004)}{-0.034}^{**}$
Some physical activity	$\underset {\phantom {-}(0.002)}{-0.020 }^{**}$	$\underset {\phantom {-}(0.003)}{-0.017 }^{**}$	$\underset {\phantom {-}(0.003)}{-0.023 }^{**}$	$\underset {\phantom {-}(0.003)}{-0.023}^{**}$	$\underset {\phantom {-}(0.002)} {-0.022}^{**}$	$\underset {\phantom {-}(0.002)}{-0.020}^{**}$	$\underset {\phantom {-}(0.002)}{\hspace {1pt} -0.017}^{**}$	$\underset {\phantom {-}(0.002)}{\hspace {.7pt}-0.017}^{**}$	$\underset {\phantom {-}(0.002)} {-0.015}^{**}$	$\underset {\phantom {-}(0.002)}{-0.015}^{**}$
Constant	$\underset {(0.008)}{\hspace {2.5pt}0.632}^{**}$	$\underset {(0.013)}{\hspace {2pt} 0.485 }^{**}$	$\underset {(0.012)}{\hspace {2.5pt}0.520 }^{**}$	$\underset {(0.012)}{\hspace {2.5pt}0.568 }^{**}$	$\underset {(0.010)}{\hspace {2.5pt}0.604}^{**}$	$\underset {(0.011)}{\hspace {2pt} 0.629 }^{**}$	$\underset {(0.009)} {\hspace {2.5pt}0.659 }^{**}$	$\underset {(0.009)}{\hspace {2pt} 0.691}^{**}$	$\underset {(0.009)}{\hspace {2pt} 0.735 }^{**}$	$\underset {(0.008)}{\hspace {2pt} 0.800}^{**}$
*R*^2^ (Pseudo *R*^2^)	0.365	0.211	0.234	0.251	0.244	0.222	0.195	0.161	0.154	0.119

*Note.* Weigthed robust standard errors are given in parentheses. ^∗^*p*<0.05; ^∗∗^*p*<0.01

## Data Availability

The dataset analysed in this study is not publicly available, but can be requested from the Melbourne Institute.

## Abbreviations

AIC: Akaike Information criterion
AUD: Australian dollar
BIC: Bayesian information criterion
CDF: Cumulative distribution function
GAM: Generalized additive models
GAMLSS: Generalized additive models for location, scale and shape
GDP: Gross domestic product
HILDA: Household, income and labour dynamics in Australia
IV: Instrumental variable
OECD: Organisation for economic co-operation and development
OLS: Ordinary least squares
Q-Q: Quantile-quantile
RIF: Recentered influence function
SF-6D: Short-form six-dimension
